# Analyzing Cytotoxic and Apoptogenic Properties of *Scutellaria litwinowii* Root Extract on Cancer Cell Lines

**DOI:** 10.1093/ecam/nep214

**Published:** 2011-03-09

**Authors:** Zahra Tayarani-Najaran, Seyed Ahmad Emami, Javad Asili, Alireza Mirzaei, Seyed Hadi Mousavi

**Affiliations:** ^1^Department of Pharmacology, School of Medicine, Mashhad University of Medical Sciences, Iran; ^2^Pharmacological Research Centre of Medicinal Plants, School of Medicine, Mashhad University of Medical Sciences, Iran; ^3^Department of Pharmacognosy, School of Pharmacy, Mashhad University of Medical Sciences, Iran; ^4^Medical Toxicology Research Center, Mashhad University of Medical Sciences, Mashhad, Iran

## Abstract

The *Scutellaria* species (Lamiaceae) is used as a source of flavonoids to treat a variety of diseases in traditional medicine. In spite of many reports about the cytotoxic and antitumor effects of some species of this genus, anticancer researches on one of the Iranian species *S. litwinowii* have not yet been conducted. The cytotoxic properties of total methanol extract of *S. litwinowii* and its fractions were investigated on different cancer cell lines including AGS, HeLa, MCF-7, PC12 and NIH 3T3. Meanwhile, the role of apoptosis in this toxicity was explored. The cells were cultured in DMEM medium and incubated with different concentrations of herb plant extracts. Cell viability was quantitated by MTT assay. Apoptotic cells were determined using propidium iodide staining of DNA fragmentation by flow cytometry (sub-G1 peak). *Scutellaria litwinowii* inhibited the growth of malignant cells in a dose-dependent manner. Among solvent fractions of *S. litwinowii*, the methylene chloride fraction was found to be more toxic compared to other fractions. The IC_50_ values of this fraction against AGS, HeLa, MCF-7 and PC12 cell lines after 24 h were determined, 121.2 ± 3.1, 40.9 ± 2.5, 115.9 ± 3.5 and 64.5 ± 3.4 **μ**g/ml, respectively. *Scutellaria litwinowii* induced a sub-G1 peak in the flow cytometry histogram of treated cells compared to control cells indicating that apoptotic cell death is involved in *S. litwinowii* toxicity. *Scutellaria litwinowii* exerts cytotoxic and proapototic effects in a variety of malignant cell lines and could be considered as a potential chemotherapeutic agent in cancer treatment.

## 1. Introduction

Plant materials have served as medicines across cultures and throughout time. Knowledge about plants that were found to be most effective against particular ailments was passed down to the succeeding generations. These caches of ancient wisdom encompassed diagnostic techniques, instructions for preparation of remedies and instructions about which herbs should be prepared with specific other natural products to achieve optimal results [1].

Herbal remedies and alternative medicines are used throughout the world and in the past herbs often represented the original sources of most drugs [2–4]. Numerous cancer research studies have been conducted using traditional medicinal plants in an effort to discover new therapeutic agents that lack the toxic side effects associated with current chemotherapeutic agents [5].

There has been a growing interest in the use of naturally occurring compounds with chemopreventive and chemotherapeutic properties for the treatment of cancers. Epidemiological studies as well as experimental approaches have revealed the anticancer properties of a multitude of medicinal herbs that are mediated through different mechanisms including altered carcinogen metabolism, induction of DNA repair systems, immune activation and suppression of cell cycle progression/induction of apoptosis. While cancer cell death/apoptosis could be considered a convergence point of all antineoplastic therapies, direct proapototic effects have been reported for bioactive phytochemicals [6].

Apoptosis is a gene-regulated phenomenon induced by many chemotherapeutic agents in cancer treatment. The induction of apoptosis in tumor cells is considered very useful in the management and therapy as well as in the prevention of cancer [7].

*Scutellaria* L. (Lamiaceae) is a genus that contains around 300 species of erect or spreading annuals, rhizomatous and clump-forming herbaceous perennials and more rarely, subshrubs, worldwide, excluding South Africa [8–10]. This genus has 20 species and two hybrids in Iran [11], in which 10 species and two hybrids are endemic to the country [12].

One species of this genus is *Scutellaria baicalensis*. This species is used as a source of flavonoids in traditional Chinese medicine. Traditionally, the dried roots of *S. baicalensis* have been used in a Chinese herbal medicine “Huang Qin” to treat a variety of diseases including viral hepatitis, inflammatory diseases, bacterial infections and a variety of tumors. Recently, it has been shown that *S. baicalensis* extracts could be useful in patients with SARS (severe acute respiratory syndrome) or SARS-like infectious diseases [13, 14]. Studies have demonstrated that flavonoids from *S. baicalensis* could arrest some tumor cell lines and inhibit tumor angiogenesis [5, 15]. *Scutellaria barbata* is another member of this family, which is also known for its antiproliferative properties [16–18].

A crude natural product extract is generally an extremely complicated mixture of several compounds that possess variable chemophysical properties. The fundamental strategy for separating these compounds is based on their chemophysical properties that can be cleverly exploited to initially separate them into various chemical groups. However, from the literature search of the related genera and families, it is possible to predict the types of compounds that might be present in a particular extract. This tentative prediction on possible identity of the classes of compounds may help choose suitable extraction and partitioning methods and solvents for extracting specific classes of compounds. Plant natural products are usually extracted with solvents of increasing polarity [19]. The larger the variety of compounds that are extracted by the extractant, the better the chance that biologically active components will also be extracted if a specific class of chemical component is not targeted [20]. In this study, the use of a solvent for screening and for the isolation of active components was examined.

*Scutellaria litwinowii* Bornm. & Sint. ex Bornm is one of the Iranian species of *Scutellaria*. Although, there are widespread reports about the cytotoxic and antitumor effects of some species of this genus, anticancer researches have not yet been conducted on *S. litwinowii*. Therefore, in an attempt it is sought to study the cytotoxic properties of *S. litwinowii* root extract on some common cancer cell lines including AGS, HeLa, MCF-7, PC12 and mouse embryo cell line (NIH 3T3) as non-malignant cells. AGS cell line was isolated from an adenocarcinoma of the stomach resected from a 54-year-old Caucasian female. In the USA, adenocarcinoma of the stomach is the seventh most common cause of cancer death [21]. HeLa cells are human epithelial cells from a fatal cervical carcinoma. The cell line was derived from cervical cancer cells taken from Henrietta Lacks, in 1951. It has been one of the most widely studied cell lines in cervical cancer, the second most frequent malignant tumor in women worldwide [22, 23]. MCF-7 is a human breast cancer cell line, which is known as a widely used model system for the study of breast cancer [24]. The PC12 cell line has been established from a transplantable rat adrenal pheochromocytoma and has been widely used to investigate neuronal cell death [25].

We also explored the role of apoptosis in *S. litwinowii*-induced cytotoxicity on cancer cell lines.

## 2. Methods

### 2.1. Reagents and Chemicals

The fluorescent probe propidium iodide (PI), sodium citrate, 3-(4,5-dimethylthiazol-2-yl)-2,5-diphenyl tetrazolium (MTT), baicalein and Triton X-100 were purchased from Sigma (St Louis, MO, USA). DMEM and FCS were purchased from Gibco (Grand Island, USA).

### 2.2. Plant Materials

**Figure 1:**
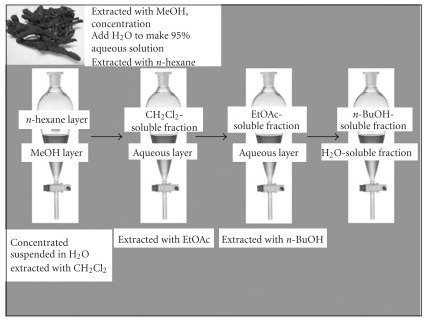
Partitioning scheme using immiscible solvents.

The roots of *S. litwinowii* were collected from Hosseinabad valley (2100 m height) in Pivejan, a village at 65 km south-west of Mashhad, Khorasan Razavi province Iran. The plant was identified by Mr M.R. Joharchi, in Ferdowsi University of Mashhad Herbarium (FUMH). Voucher specimen was deposited in herbarium of faculty of pharmacy, University of Mashhad Medical Sciences. Dry powdered roots (100 g) of *S. litwinowii* were extracted with methanol (4 × 0.5 l) and were then concentrated at 50°C under reduced pressure to dryness. The concentrated extract was then extracted with an equal volume of *n*-hexane, three times, to give a fraction containing non-polar compounds, such as lipids. The process is referred to as defatting. Then the solution was successively partitioned between CH2Cl2, ethylacetate (EtOAc) and *n*-butanol (*n*-BuOH) and finally water. The isolated fractions were also dried. A partitioning scheme of the methanol extract of *S. litwinowii* is presented in Figure 1 [19].

All the isolated fractions were subjected to cytotoxic and apoptosis assays.

### 2.3. Cell Culture

AGS, HeLa, MCF-7, PC12 and NIH 3T3 were obtained from Pasteur Institute (Tehran, Iran) and maintained at 37°C in a humidified atmosphere (90%) containing 5% CO_2_. Cell lines were cultured in Dulbecco's modified Eagle's medium (DMEM) with 5% (v/v) fetal bovine serum, 100 U ml^−1^ penicillin and 100 *μ*g ml^−1^ streptomycin. Cells were seeded overnight and then incubated with various concentrations of different extracts for 24 and 48 h.

For MTT assay, cells were seeded at 5000 cell per well onto 96-well culture plates. For assay of apoptosis, cells were seeded at 100 000 cell per well onto a 24-well plate. For each concentration and time course study, there was a control sample that remained untreated and received the equal volume of medium.

### 2.4. Cell Viability

Cell viability was determined using a modified MTT assay [26, 27]. Briefly, the cells were seeded (5000 cell per well) onto flat-bottomed 96-well culture plates and allowed to grow for 24 h followed by treatment with total extract (5–1000 *μ*g ml^−1^), *n*-Hexane (5–160 *μ*g ml^−1^), defatted fraction (MeOH layer in Figure 1) (6.25–1280 *μ*g ml^−1^), CH_2_Cl_2_, EtOAc, *n*-BuOH, H_2_O soluble fractions (5–160 *μ*g ml^−1^ of each fraction). After removing the medium, the cells were labeled with MTT solution (5 mg ml^−1^ in PBS) for 4 h and the resulting formazan was solubilized with DMSO (100 *μ*l). The absorption was measured at 570 nm (620 nm as a reference) in an ELISA reader. The most sensitive cell lines were compared with baicalein as a positive control.

### 2.5. Apoptosis

Apoptotic cells were detected using PI staining of treated cells followed by flow cytometry to detect the so-called sub-G1 peak [27, 28]. It has been reported that DNA fragmentation creates small fragments of DNA that can be eluted following incubation in a hypotonic phosphate-citrate buffer. When stained with a quantitative DNA-binding dye such as PI, cells that have lost DNA will take up less stain and will appear to the left of the G1 peak. Briefly, HeLa cells were cultured overnight in a 24-well plate and treated with *S. litwinowii* for 24 h. Floating and adherent cells were then harvested and incubated at 4°C overnight in the dark with 750 *μ*l of a hypotonic buffer (50 *μ*g ml^−1^ PI in 0.1% sodium citrate + 0.1% Triton X-100) before flow cytometric analysis using a FACScan flow cytometer (Becton Dickinson) was conducted. Ten thousand events were acquired with FACS.

### 2.6. Statistics

One-way analysis of variance (ANOVA) and Bonferroni's *post hoc* were used for data analysis. All results were expressed as mean ± SD. *P *< .05 were considered statistically significant.

## 3. Results

### 3.1. Inhibition of Cell Viability

#### 3.1.1. Total Methanol Extract of *S. litwinowii*

**Figure 2:**
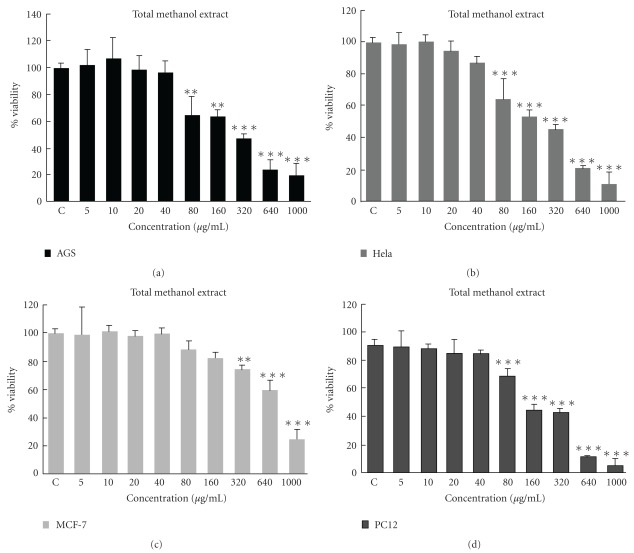
Dose-dependent growth inhibition of malignant cell lines by total methanol extract (5–1000 *μ*g ml^−1^) after 48 h. Viability was quantitated by MTT assay. The toxicity started at a concentration as little as 80 *μ*g ml^−1^ and the dose inducing IC_50_ against AGS, HeLa, MCF-7 and PC12 was calculated 216.1, 250.0, 723 and 243.9, respectively. Results are mean ± SD (*n* = 3).**P < .01 and ***P < .001 compared to control.

The cytotoxicity of total methanol extract of *S. litwinowii* and its different fractions were examined on malignant cell lines. First, malignant cells were incubated with various concentrations of total methanol extract of *S. litwinowii* (5–1000 *μ*g ml^−1^) for 48 h. The result showed this extract decreased cell viability of cells in a concentration-dependent manner. The toxicity started at a concentration as little as 80 *μ*g ml^−1^ and the dose inducing 50% cell growth inhibition (IC_50_) against AGS, HeLa, MCF-7 and PC12 was calculated 216.1, 250.0, 723 and 243.9, respectively (Figure 2).

#### 3.1.2. Defatted Fraction of *S. litwinowii*

**Figure 3:**
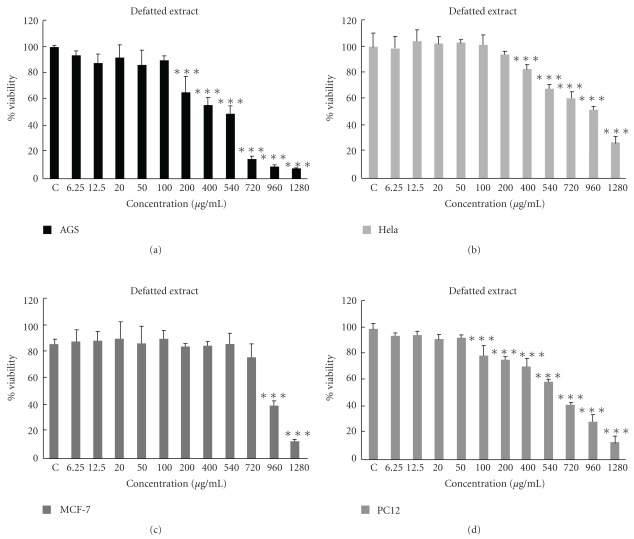
Dose-dependent growth inhibition of malignant cell lines by defatted fraction (6.25–1280 *μ*g ml^−1^) after 24 h. Viability was quantitated by MTT assay. The IC_50_ of defatted extract against AGS, HeLa, MCF-7 and PC12 was 372.0, 888.1, 1150 and 567.5 for 24 h, respectively. Results are mean ± SD (*n* = 3). ***P < .001 compared to control.

Our findings about the defatted fraction of *S. litwinowii* (6.25–1280 *μ*g ml^−1^) also showed antiproliferative effects on these cell lines (Figure 3). The IC_50_ of the defatted extract against AGS, HeLa, MCF-7 and PC12 was 372.0, 888.1, 1150 and 567.5 for 24 h, respectively.

#### 3.1.3. CH_2_Cl_2_ Fraction of S. litwinowii

**Figure 4:**
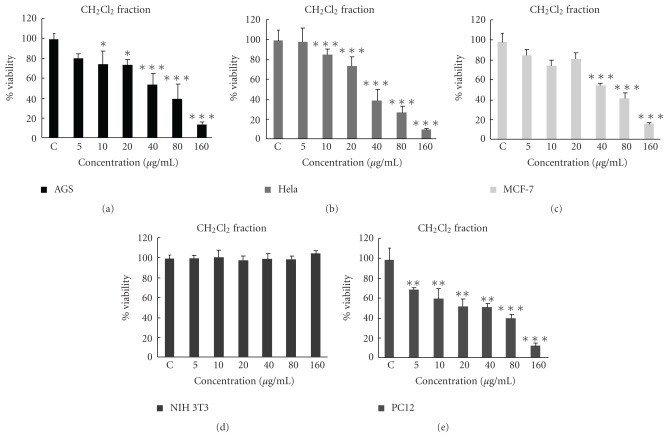
Dose-dependent growth inhibition of malignant cell lines by CH_2_Cl_2_ fraction (5–160 *μ*g ml^−1^) after 24 h. Viability was quantitated by MTT assay. The IC_50_ values of this fraction against AGS, HeLa, MCF-7 and PC12 cell lines after 24 h were determined, 121.2 ± 3.1, 40.9 ± 2.5, 115.9 ± 3.5 and 64.5 ± 3.4 *μ*g ml^−1^, respectively. Results are mean ± SD (*n* = 3). *P < .05, **P < .01 and ***P < .001 compared to control.

In order to compare the cytotoxicity of solvent fractions of *S. litwinowii* against malignant cells, another MTT assay was carried out at different concentrations (5–160 *μ*g ml^−1^). Among them, the CH_2_Cl_2_ fraction was found to be more effective than the other fractions of the plant (Figure 4), whereas the other fractions showed no prominent cytotoxicity on the cell lines tested (Table 1). The CH_2_Cl_2_ fraction showed inhibitory effect on the proliferation of malignant but not non-malignant cells indicating a degree of specificity for malignant cell lines. The IC_50_ values of this fraction against AGS, HeLa, MCF-7 and PC12 cell lines after 24 h were determined, 121.2 ± 3.1, 40.9 ± 2.5, 115.9 ± 3.5 and 64.5 ± 3.4 *μ*g ml^−1^, respectively (Table 1).

**Figure 5:**
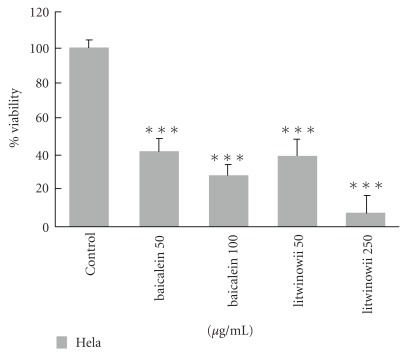
Growth inhibition of HeLa cells by CH_2_Cl_2_ fraction (50 and 250 *μ*g ml^−1^) compare with baicalein (50 and 100 *μ*m) after 24 h. Viability was quantitated by MTT assay. CH_2_Cl_2_ fraction could inhibit the proliferation of cells that is comparable with baicalein. Results are mean ± SD (*n* = 3). ***P < .001 compared to control.

HeLa cells, as the most sensitive cell line, were selected for further comparative studies with baicalein as a positive control [15]. The CH_2_Cl_2_ fraction could inhibit the proliferation of cells in a manner that is comparable with baicalein Figure 5. 

### 3.2. Apoptosis

**Figure 6:**
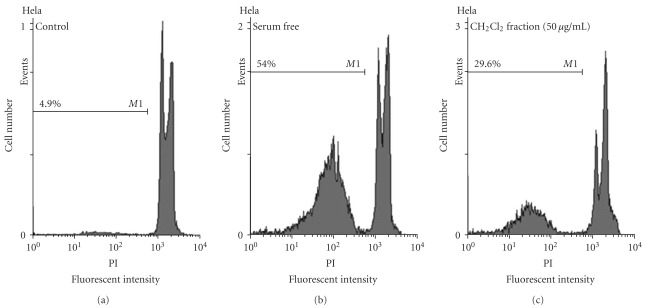
Flow cytometry histograms of apoptosis assays by PI method in HeLa cells. Cells were treated with 50 *μ*g ml^−1^ of CH_2_Cl_2_ fraction for 24 h. Sub-G1 peak as an indicative of apoptotic cells, was induced in CH_2_Cl_2_ fraction treated but not in control cells. (1) control, (2) serum free (positive control); and (3) CH_2_Cl_2_ fraction. Flow cytometry histogram of positive control in which cells were cultured in serum free medium and CH_2_Cl_2_ fraction-treated cells. CH_2_Cl_2_ fraction-treated cells exhibited a sub-G1 peak in HeLa cells that indicates the involvement of an apoptotic process in CH_2_Cl_2_ fraction-induced cell death.

Apoptosis following treatment with CH_2_Cl_2_ fraction of *S. litwinowii* (50 *μ*g ml^−1^) was measured with PI staining and flow cytometry, aiming to detect the sub-G1 peak resulting from DNA fragmentation. Flow cytometry histogram of the positive control in which cells were cultured in serum free medium [29] and of CH_2_Cl_2_ fraction-treated cells were studied. CH_2_Cl_2_ fraction-treated cells exhibited a sub-G1 peak in HeLa cells that indicates the involvement of an apoptotic process in CH_2_Cl_2_ fraction-induced cell death (Figure 6).

## 4. Discussion

Natural products have long been used to prevent and treat diseases including cancers and might be good candidates for the development of anticancer drugs.

In this study, the cytotoxic and proapoptotic effects of *S. litwinowii* on different cancer cell lines were investigated. To the authors knowledge this is the first report on *S. Litwinowii*-induced toxicity in cancer cell lines. Our data confirmed that *S. litwinowii* extract has cytotoxic activity against AGS, HeLa, MCF-7 and PC12 cell lines, which is consistent with previous studies conducted on other species of *Scutellaria* genus. Different studies have shown the antiproliferative activity of *Scutellaria* species including *S. baicalensis* and *S. barbata* [5, 15–18, 30].

In this study, the purification by solvent extraction of *S. litwinowii* was used and the potential antitumor activity of low-polar solvent fractions (*n*-Hexane, CH_2_Cl_2_, EtOAc) was compared to polar solvent fractions (*n*-BuOH and H_2_O soluble). It was found that CH_2_Cl_2_ fraction had the greatest antiproliferative activity *in vitro*. The effect of CH_2_Cl_2_ fraction on non-malignant cells showed a degree of specificity for malignant cell lines. It has also been found that among different fractions of the studied *Scutellaria* genus, the methylene chloride and chloroform fractions were more effective than other fractions [31–34].

Other species of studied *Scutellaria* contains three major flavonoids including baicalin, baicalein and wagonin, whose cytotoxic properties against different cancer cell lines have been shown previously [15, 35]. Similarly, in our study, the cytotoxic and apoptogenic properties of *S. litwinowii* root extract could also be attributed to these flavonoids.

**Figure 7:**
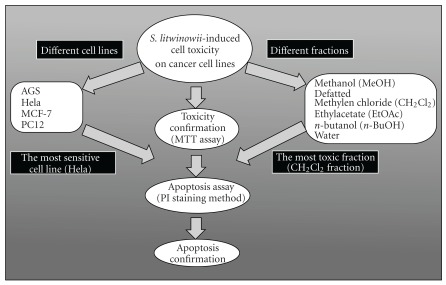
Approach to analyzing cytotoxic and apoptogenic properties of *S. litwinowii* root extract on cancer cell lines.

In the present study, *S. litwinowii*-induced apoptosis was also shown to be involved in the induction of cell death in the HeLa cell line (Figure 7). Apoptotic cell death is known to be induced by many chemotherapeutic agents routinely used in cancer treatment regimens. Apoptosis is characterized by distinct morphological features including, chromatin condensation, cell and nuclear shrinkage, membrane blebbing and oligonucleosomal DNA fragmentation. Apoptosis is an important homeostatic mechanism that balances cell division and cell death and maintains the appropriate number of cell in the body. In the present study, apoptosis was determined using PI staining of DNA fragmentation by flow cytometry (sub-G1 peak). It has been reported that DNA fragmentation creates small fragments of DNA that can be eluted following incubation in a hypotonic phosphate-citrate buffer. When stained with a quantitative DNA-binding dye such as PI, cells that have lost DNA will take up less stain and will appear to the left of the G1 peak [27, 28]. A balance between cell proliferation and apoptosis controls normal organ development [36–38]. The induction of apoptosis in tumor cells is considered a valuable way to treat cancer [39]. A wide variety of natural substances have been recognized to have the ability to induce apoptosis in various tumor cells. It is thus considered important to screen apoptotic inducers from plants, either in the form of crude extracts or as components isolated from them [6].

In this study baicalein could inhibit the proliferation of HeLa cells. Baicalein is a flavone isolated from the plant *Scutellariae radix*, which is commonly used as a dietary supplement in Asian countries. This flavonoid has modulating effects on drug metabolizing enzymes and antiproliferative effects on cancer cells [40].

Taking together, this study showed that *S. litwinowii* inhibits the proliferation of a variety of malignant cell lines with the involvement of apoptosis or programmed cell death.

Further studies are needed to fully recognize the mechanisms involved in cell death. *Scutellaria litwinowii* could also be considered as a promising chemotherapeutic agent in cancer treatment.

## Figures and Tables

**Table 1:** Doses inducing IC_50_ of solvent fractions of *S. litwinowii* against malignant cell lines.

Cell line	Fraction				
	n-Hexane	CH_2_Cl_2_	EtOAc	*n*-BuOH	H_2_O
AGS	>300	121.2 ± 3.1	>300	>300	>300
HeLa	>300	40.9 ± 2.5	>300	>300	>300
MCF-7	>300	115.9 ± 3.5	>300	>300	>300
PC12	>300	64.5 ± 3.4	>300	>300	>300

Cells incubated with different concentration of extracts for 24 h. IC_50_ values were expressed as the mean ± SD (*n* = 3).
